# Induction of left ventricular hypoplasia by occluding the foramen ovale in the fetal lamb

**DOI:** 10.1038/s41598-020-57694-4

**Published:** 2020-01-21

**Authors:** Flora Y. Wong, Alex Veldman, Arun Sasi, Mark Teoh, Andrew Edwards, Yuen Chan, Oliver Graupner, Christian Enzensberger, Roland Axt-Fliedner, Mary Jane Black, Dietmar Schranz

**Affiliations:** 1 The Ritchie Centre, Hudson Institute of Medical Research, Monash University, Melbourne, Australia; 2 Monash Newborn, Monash Medical Centre, Melbourne, Australia; 3 Department of Pediatrics, Monash University, Melbourne, Australia; 4 Pediatric Heart Center, Justus-Liebig University, Giessen, Germany; 5 Department of Pediatrics, St. Vincenz Hospital, Limburg, Germany; 6 Perinatal Services, Monash Medical Centre, Melbourne, Australia; 7 Department of Pathology, Monash Medical Centre, Melbourne, Australia; 8 Department of Obstetrics and Gynecology, University Hospital Giessen and Marburg (UKGM), Giessen, Germany; 9 Department of Anatomy and Developmental Biology, Monash University, Melbourne, Australia

**Keywords:** Heart development, Interventional cardiology, Congenital heart defects

## Abstract

Disturbed fetal haemodynamics often affects cardiac development and leads to congenital cardiac defects. Reduced left ventricular (LV) preload in the fetus may result in hypoplastic LV, mitral and aortic valve, mimicking a moderate form of hypoplastic left heart complex. We aimed to induce LV hypoplasia by occluding the foramen ovale (FO) to reduce LV preload in the fetal sheep heart, using percutaneous trans-hepatic catheterisation. Under maternal anaesthesia and ultrasound guidance, hepatic venous puncture was performed in six fetal lambs (0.7–0.75 gestation). A coronary guidewire was advanced into the fetal inferior vena cava, right and left atrium. A self-expandable stent was positioned across the FO. An Amplatzer Duct Occluder was anchored within the stent for FO occlusion. Euthanasia and post-mortem examination was performed after 3 weeks. Nine fetuses were used as age-matched controls. Morphometric measurements and cardiac histopathology were performed. Compared with controls, fetal hearts with occluded FO had smaller LV chamber, smaller mitral and aortic valves, lower LV-to-RV ratio in ventricular weight and wall volume, and lower number of LV cardiomyocyte nuclei. We conclude that fetal FO occlusion leads to a phenotype simulating LV hypoplasia. This large animal model may be useful for understanding and devising therapies for LV hypoplasia.

## Introduction

The heart is the first organ to function during embryonic development, and first contractions of the primitive heart tube are observed as early as from 2 weeks of gestation onwards. Consequently, almost all stages of heart development and growth take place in a working heart with active blood flow. Haemodynamic forces are critical for normal cardiac development, and interference in haemodynamics often leads to congenital heart defects^1^.

Experimental manipulation of flow through developing left-sided cardiac structures exerts profound effects on left atrium (LA), left ventricle (LV), and mitral and aortic valve development. Already in 1973, Harth *et al*. described the induction of hypoplastic left heart complex (HLHC) in chick embryos by obstruction of blood flow through the left atrioventricular canal^2^. A similar effect was observed by Fishman *et al*. 5 years later in fetal sheep when inserting a balloon in the LA obstructed LV filling and produced the phenotype of HLHC, however all of the fetal lambs died within a few days of the procedure^3^. Others described induction of a HLHC by reducing LV filling due to LA ligation in the chick embryo^4,5^. Interestingly, such induced LV hypoplasia can be rescued by an increased volume loading of the left heart at a later timepoint^6^.

In the fetus, LA and LV filling is almost completely dependent on right to left shunting of oxygenated blood from the placenta through the foramen ovale (FO) since the right ventricular (RV) output is mostly bypassing the lungs through the ductus arteriosus and LA filling by pulmonary venous return is only marginal. Premature closure or restriction of the FO, eliminating or restricting the fetal right to left atrial shunt, has been described to have variable consequences, probably depending on the timing and the presence or absence of accompanying cardiac malformations. Several authors have reported RV to LV growth discrepancy and mild to moderate hypoplasia of the mitral and aortic valves as well as the aortic arch^7,8^, others described LV dilatation and fibro-elastosis^9^. While RV dilation, tricuspid regurgitation, pericardial effusion, pleural effusion, in-utero heart failure and hydrops fetalis as well as early and late pulmonary hypertension have also been reported^10–14^, there is substantial evidence that early (primary) closure of the FO results in hypoplasia of left heart structures and that normal fetal left heart development is dependent on a normal volume of flow though the FO^9,15^.

Here, we report the effects of in-utero occlusion of the FO in the fetal lamb in mid-gestation, using a novel fetal cardiac catheterisation method with trans-hepatic access that we have described previously^16,17^. Our technique of in-utero cardiac catheterisation allows occlusion of the FO without trauma or puncture into the fetal heart, and study of the effects of FO occlusion during fetal development. We hypothesized that by reducing volume loading of the left sided heart structures, a fetus with obliterated or obstructed blood flow through the FO would develop the phenotype of HLHC.

## Results

### Technical success and failure

**Figure 1:**
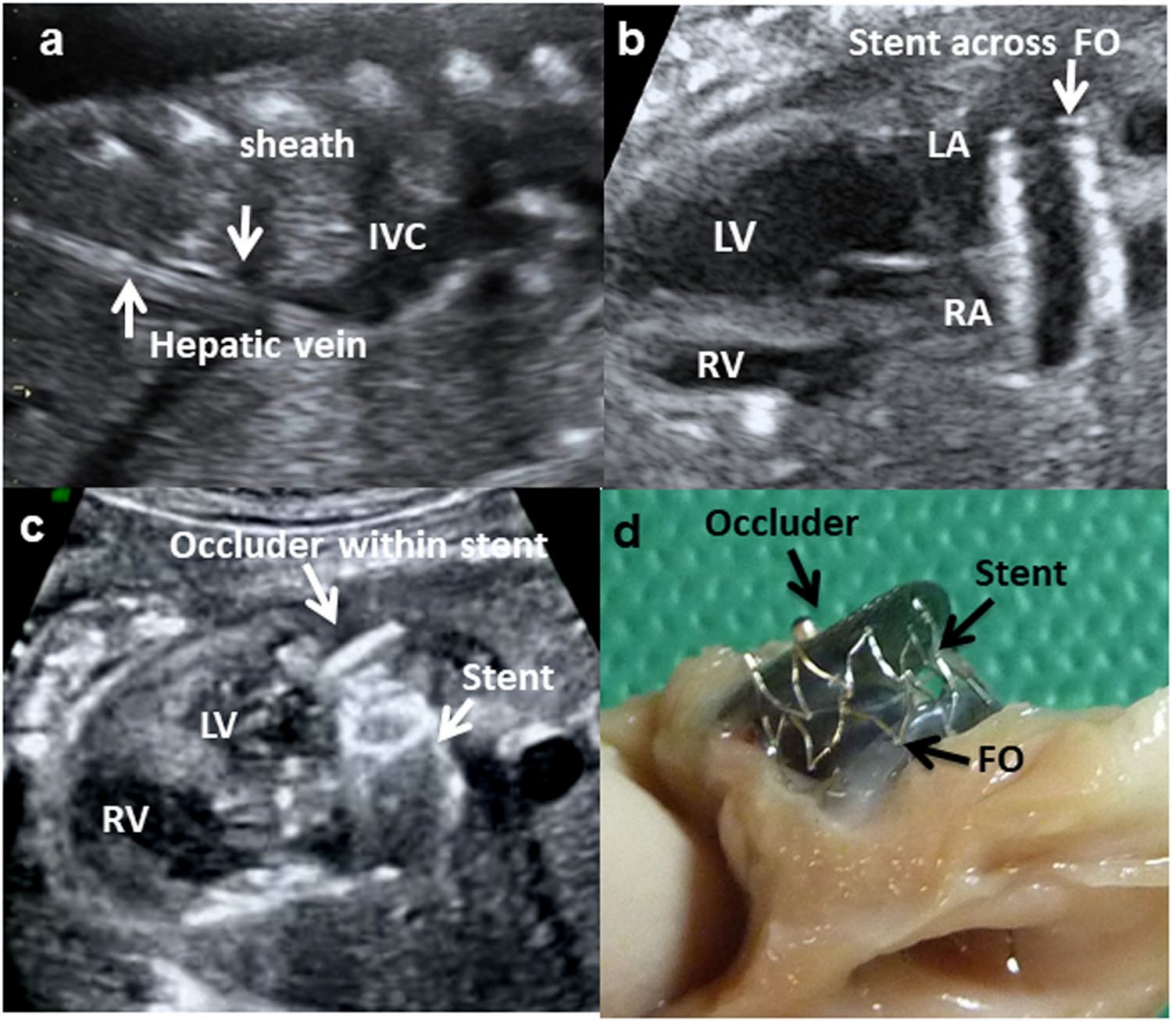
Ultrasound images of the fetal procedure to occlude the foramen ovale (FO): (**a**) the cannula sheath extending along the fetal hepatic vein into the inferior vena cava (IVC), (**b**) a stent is positioned across the FO, and (**c**) the occluder is anchored within the stent. The left ventricle (LV) has collapsed with FO occlusion. (**d**) Post-mortem examination shows lateral view of the occluder anchored within a stent placed across the FO.

Total of 26 fetuses had the transhepatic catheterisation. Access to the fetal venous system, followed by passage of catheter and guidewire though the RA and FO into the LA was successfully in all animals. Anchoring the occluder within the stent at FO was successful in 16 out of 26 fetuses. The 16 animals were then monitored with fetal ultrasounds regularly until the planned euthanasia at least 3 weeks later. Two fetuses died within 1–2 days after the procedure, with intra-abdominal haemorrhage found at post-mortem examination. One fetus died the day before the planned euthanasia, and post-mortem examination revealed the stent and occluder had dislodged into the mitral valves. On post-mortem examination after the planned euthanasia, the occluder was found to be in various oblique positions within the stent and even partly in the left atria in 7 animals, and therefore did not effectively obliterate FO blood flow. These 7 fetuses had variable structural cardiac changes from the partial FO occlusion (Supplementary Table S1) and they were therefore excluded from cardiac histopathology analyses. Six fetuses had successful occlusion of the FO confirmed on post-mortem examination (Fig. 1d).

For these 6 fetuses, averaged time in-utero between the FO occlusion and euthanasia was 25.5 days (Table 1). Through the course of regular fetal ultrasound monitoring during that period, none of the animals showed pleural or pericardial effusions. Hydrops fetalis or fetal compromise was not observed. Averaged gestational age at euthanasia of the 6 fetuses was 135.8 days. Nine age-matched fetuses were used as controls (Table 1).

**Table 1:** Animal Demographics.

Fetuses with occluded FO	sex	Age at stent/occluder insertion	Age at post-mortem
1*	M	110	131
2*	M	111	131
3*	M	117	137
4*	M	114	139
5	M	103	138
6	F	104	139
**Controls**	**sex**		**Age at post-mortem**
1*	M		131
2*	M		131
3*	M		131
4*	F		137
5*	M		137
6*	M		137
7*	M		139
8	M		138
9	F		139

*Animals which had cardiac histopathology performed.

### Ultrasonographic measurements

For the 6 fetuses with successful FO occlusion, ultrasonographic measurements before the fetal transhepatic catheterisation showed that their RHV and LHV had mean (SD) diameters of 5.4 (0.7) mm and 4.7 (0.8) mm respectively. The distances along the IVC from the RHV and LVH to the right atrium were 32.8 (3.9) mm and 32.4 (3.8) mm respectively. The IVC and FO had a diameter of 7.2 (0.8) mm and 6.5 (0.9) mm respectively. Other intra-cardiac measurements are shown in Table 2.

**Table 2:** Fetal measurements on ultrasonography before and after occlusion of the foramen ovale (n = 6).

Tricuspid valve (mm)	Mitral valve (mm)	Pulmonary valve (mm)	Aortic valve (mm)	RV width (mm)	LV width (mm)
**Pre-occlusion**					
9.8 (0.4)	10.3 (1.6)	8.8 (0.8)	7.1 (1.1)	10.2 (2.4)	11.5 (2.1)
**Post-occlusion**					
9.6 (1.5)	8.4 (1.3) *	8.4 (1.8)	6.2 (1.2)	10.9 (3.0)	9.4 (2.9)*

Values are mean (SD). *P < 0.05 compared to pre-occlusion value.

**Figure 2:**
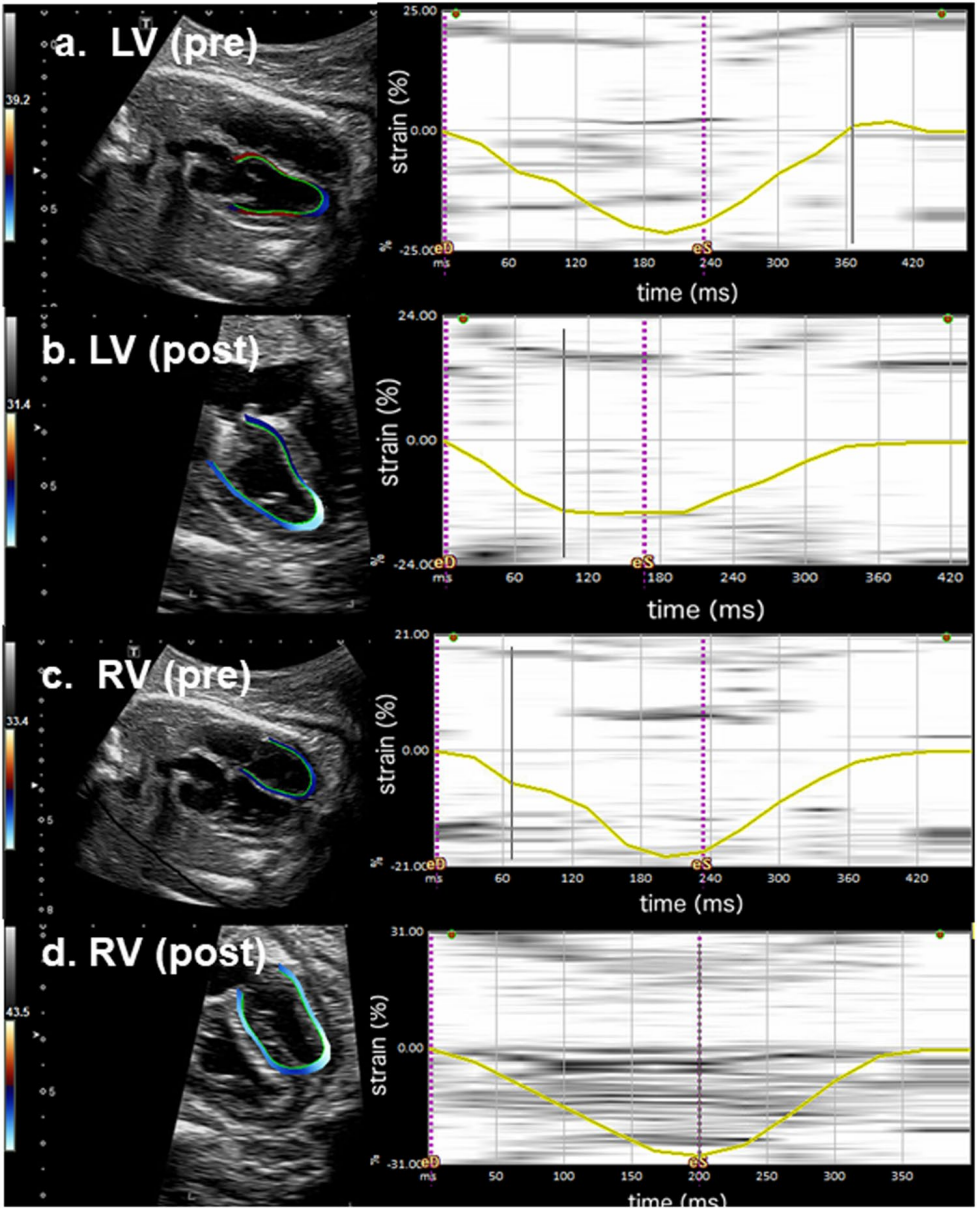
Speckle tracking echocardiography: Left panels-Speckle tracking echocardiography of the fetal lamb with 4-chamber-view and traced endocardial border of the ventricle. Right panels-Graphically displayed global longitudinal peak systolic strain (LPSS, yellow line) of one cardiac cycle; dotted purple line marking end-diastole (eD) and end-systole (eS). (**a**) Normal left-ventricular global myocardial deformation pattern pre-occlusion of the FO (LV pre) with global LPSS of −19.7%. (**b**) Post FO occlusion (LV post), the global LPSS is markedly reduced to −14.1%. (**c**) Normal right-ventricular global myocardial deformation pattern pre-occlusion of the FO (RV pre) with global LPSS of −18.6%. (**d**) Post FO occlusion (RV post), immediate increase of RV global LPSS to −29.3%.

After the FO occlusion, ultrasonographic measurements showed significantly reduced diameters of the mitral valve and width of the left ventricle (Table 2, Fig. 1c). The strain analyses in one fetus showed the mean LV and RV global LPSS was −19.7% and −18.6% respectively before the FO occlusion. The LV LPSS reduced to −14.1% immediately after the FO occlusion and −12.33% the day after. The mean RV LPSS increased to −29.3% immediately after the procedure but returned to −19.8% the day after (Fig. 2). In its control fetus, the mean LV and RV global LPSS was −18.2% and −17.2% at baseline. These values remained similar at end of anaesthesia (LV: −17.4%; RV: −19.5%) and the day after (LV: −17.5%, RV: −16.4%).

### Cardiac morphometry

**Figure 3:**
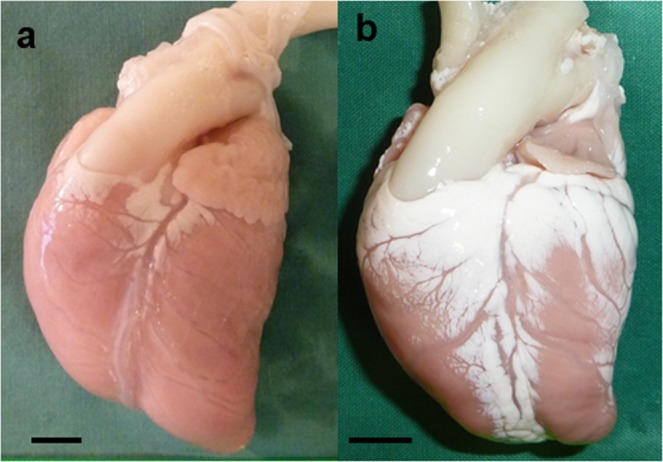
Post-mortem examination: The fetal heart with occluded FO (**b**) had a smaller LV apex compared to the control (**a**). Scale bar = 1 cm.

**Figure 4:**
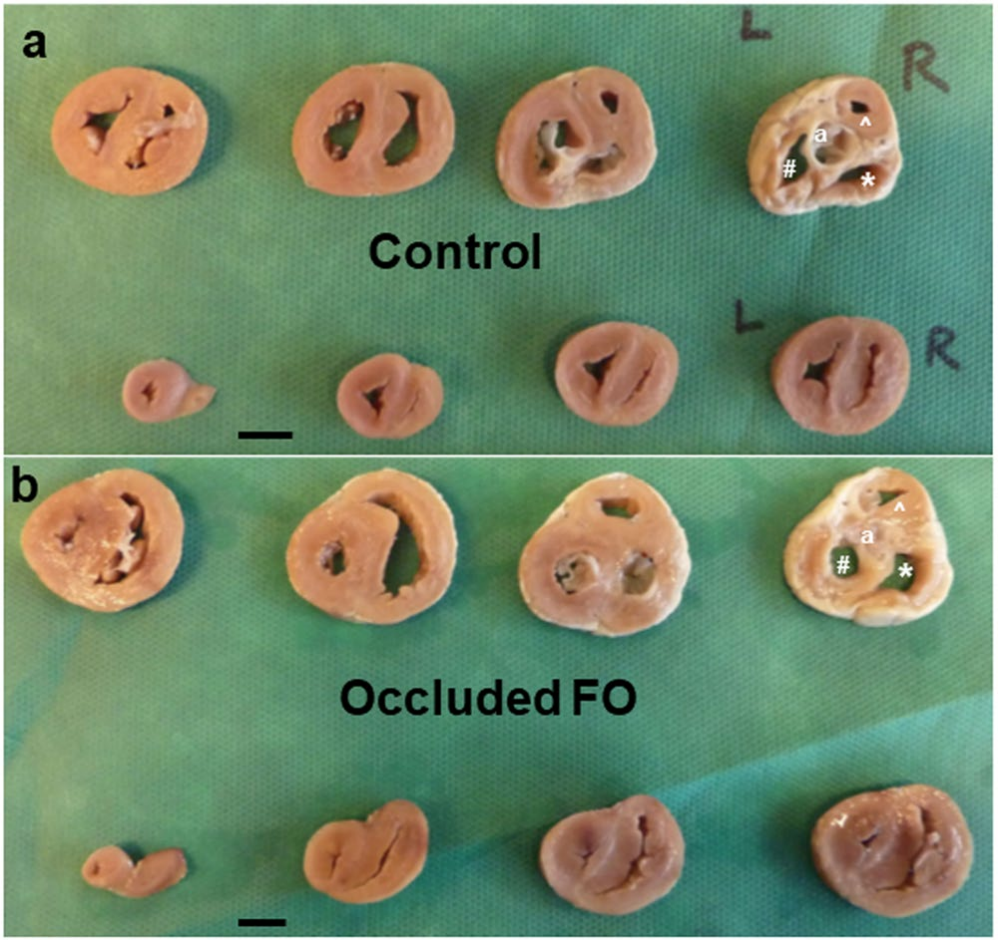
Transverse slices of the fetal ventricles at 5 mm thickness: The first slice is at the level of aortic valves (far right in upper row). The fetal heart with occluded FO (**b**) has reduced lumen of the left ventricle (LV), and smaller LV apex (last slice, far left in lower row), compared to the control (**a**). ^a^Aortic valves, ^#^LV, *RV, ^RV outflow tract. Scale bar = 1 cm.

External examination showed that the fetal hearts with occluded FO had a smaller LV apex compared to the control (Fig. 3). Body weight, weight of both ventricles and the ratio of ventricle-to-body weight were not different between animals with occluded FO and the controls (Table 3). Fetuses with occluded FO showed significant lower cross diameter of the aortic valve, with a trend towards smaller mitral valvular area and significantly lower ratio of mitral valve-to-tricuspid valvular area (Table 3 and Fig. 4).

**Table 3:** Cardiac Morphology.

Values are mean (SD)	Control (n = 9)	Occluded foramen ovale (n = 6)	P
Body weight, g	5233 (65)	4853 (48)	0.25
Weight of both ventricles, g	23.61 (5.7)	21.13 (3.2)	0.35
Ratio of ventricle to body weight	4.5 (0.7) × 10^−4^	4.3 (0.4) × 10^−4^	0.70
Aortic valve cross-diameter, mm	8.9 (1.6)	4.8 (1.0)	**<0.001**
**Atrio-ventricular valve area, cm**^**2**^			
Mitral valve (MV)	1.17 (0.45)	0.72 (0.31)	0.06
Tricuspid valve (TV)	0.85 (0.37)	0.83 (0.40)	0.16
MV/TV ratio	1.44 (0.28)	0.88 (0.09)	**<0.001**
**Ventricular chamber volume, ml**			
LV	2.28 (1.53)	0.92 (0.72)	0.06
RV	2.27 (1.45)	2.33 (1.57)	0.94
LV/RV ratio	1.00 (0.14)	0.39 (0.14)	**0.002**
**Ventricle weight, g**			
LV + S	12.97 (3.34)	11.08 (2.25)	0.25
RV	6.61 (1.77)	8.08 (1.43)	0.11
LV + S/RV ratio	1.98 (0.18)	1.37 (0.12)	**<0.001**
**Ventricle wall volume, cm**^3^			
LV + S	13.98 (3.77)	11.00 (0.85)	0.08
RV	6.44 (1.33)	7.57 (0.91)	0.09
LV + S/RV ratio	2.16 (0.24)	1.47 (0.16)	**<0.001**

Fetuses with occluded FO had a lower LV chamber volume with significantly lower LV to RV chamber volume ratio compared to controls (Table 3 and Fig. 4). While there was no significant difference between the 2 groups in the weights of the LV + S and the RV respectively, fetuses with occluded FO had a significant lower ratio of LV + S to RV weight (Table 3). A similar pattern was observed for ventricle wall volume, with the fetuses with occluded FO having a significantly lower LV + S to RV wall volume ratio compared to the controls (Table 3).

Cardiac morphometry data of the fetuses with partially occluded FO due to suboptimal and variable occluder positions are shown in Supplementary Table S1.

### Cardiac histopathology

Cardiac histopathology was performed in 4 fetuses with occluded FO and 7 age-matched controls (Table 1).

#### Cardiomyocyte number

Compared to the controls, animals with occluded FO had significantly lower LV + S cardiomyocyte nuclei count after adjusting for biventricular weight and, accordingly, the LV + S to RV ratio of cardiomyocyte nuclei count was significantly lower in animals with occluded FO compared to controls (Table 4).

**Table 4:** Cardiomyocyte nuclei counts (NC) and cell size.

Values are mean (SD)	Control (n = 7)	Occluded foramen ovale (n = 4)	P
**Cardiomyocyte NC**			
LV/RV ratio of cardiomyocyte NC	1.79	1.38	**0.02**
LV cardiomyocyte NC/biventricular wt, ×10^9^/g	0.79 (0.12)	0.59 (0.09)	**0.02**
RV cardiomyocyte NC/biventricular wt, ×10^9^/g	0.45 (0.10)	0.43 (0.08)	0.67
LV cardiomyocyte NC/LV wt, ×10^9^/g	1.44 (0.29)	1.09 (0.09)	0.053
RV cardiomyocyte NC/RV wt, ×10^9^/g	1.66 (0.37)	1.10 (0.20)	**0.02**
LV/RV density (NC/own ventricular wt) ratio	0.88 (0.15)	1.01 (0.1)	0.18
**Cell size of mono- and bi-nucleated cardiomyocytes**			
LV binucleated cardiomyocyte size, µm^2^	1783.6 (91.9)	1844.4 (71.7)	0.28
RV binucleated cardiomyocyte size, µm^2^	1727.6 (63.9)	1749.8 (49.7)	0.57
LV mononucleated cardiomyocyte size, µm^2^	917.3 (80.0)	929.7 (50.2)	0.79
RV mononucleated cardiomyocyte size, µm^2^	836.4 (98.5)	880.0 (64.4)	0.45

wt: weight.

After adjusting for individual ventricular weight (Table 4), fetuses with occluded FO showed lower densities of cardiomyocyte nuclei in both ventricles which reached statistical significance for the RV (RV: p = 0.02, LV + S: p = 0.053).

#### Cardiomyocyte size

There was no significant difference between fetuses with occluded FO and the controls in the longitudinal cross-sectional area of cardiomyocytes in the LV + S and RV (Table 4).

#### Interstitial fibrosis

There was no significant difference between fetuses with occluded FO and the controls in the level of cardiac interstitial fibrosis in the LV + S (1.8 ± 1.3% vs 3.0 ± 1.4%), RV (2.2 ± 1.1% vs 2.6 ± 1.2%) or LV + S to RV fibrosis ratio (1.1 ± 0.8 vs 1.2 ± 0.4) respectively.

## Discussion

Based on our previous experience of fetal cardiac interventions in a sheep model^16–18^, we present here a further novel interventional technique, which to our knowledge has not been performed before. We have previously described that stenting of a large FO in a sheep fetus seems to be possible only by a specially designed self-expandable stent which is deliverable through a 4 F sheath but expands to reach a stent lumen width of 8 mm^17^. The 8-mm stent lumen, together with its additional open-cell design, enables secure fixation in a widely opened FO with floppy atrial septum tissue around. Furthermore, the secured stent allows anchorage of a nitinol occluder to occlude the FO and create a model to induce the fetal HLHC. The interventional techniques and devices used in this study, all certified with the CE-mark for human use, could possibly be applied in treating other fetal cardiac malformations in future.

Our study showed that FO occlusion in fetal sheep at 0.70–0.75 gestation led to reduced LV and increased RV strain on echocardiography, indicating altered ventricular mechanics in the context of acutely modified haemodynamics of both ventricles. Within 3–4 weeks the FO occlusion produced a phenotype of LV hypoplasia with smaller LV chamber volume, small but morphologically normal mitral and aortic valves, lower LV-to-RV ratio in ventricular weight and wall volume. The significant lower LV-to-RV ratios in weight and wall volume after FO occlusion suggest that the weight and wall volume were mildly reduced for the LV, but mildly increased for the RV, though the individual RV and LV measurements did not reach statistical significance when compared with the controls. The findings imply a combination of LV hypoplasia and RV hypertrophy. This phenotype bears a striking resemblance to a report in human babies born with closed FO, hypoplasia of LV, reduced mitral and aortic valve area as well as RV hypertrophy and dilatation^8^. At the cellular level, the significantly lower LV-to-RV ratio of cardiomyocytes and reduced LV cardiomyocytes per biventricular weight in the fetuses with occluded FO also suggested LV hypoplasia with reduced cardiomyocyte proliferation. Interestingly, these animals showed reduced RV cardiomyocyte density when corrected for the slightly increased right ventricular weight, suggestive of either RV cardiomyocyte hypertrophy and/or increased fibrosis. However, we did not identify any difference in cardiomyocyte cross-sectional area or percentage of cardiac fibrosis in comparison to the controls. Notably, the cardiomyocyte area was a 2D measurement and may not represent the cell volume, and that acquired cardiac fibrosis is a rare phenomenon in fetal as well as neonatal heart^19^.

One might speculate that an isolated occlusion of the FO without additional restrictive lesions would affect mostly the development of left heart structures as a result of poor LA and LV filling, whereas additional restriction on either the RV outflow or the ductal level might favor a phenotype of RV dilation, tricuspid regurgitation and hydrops fetalis^20–22^. *Nowlen et al*. proposes that early (primary) closure of the FO, due to abnormal development of the atrial septum, results in LV hypoplasia^9^ and that late FO closure is more of a secondary event to aortic stenosis and LV dilation. Considering the “chicken and egg” phenomenon, we agree that a secondary closure of the FO has to be differentiated from primary FO closure or obstruction of blood flow through the FO. However, our work shows that even late primary cessation of FO flow is disruptive to LV growth.

Notably, in a study on prenatal narrowing or closure of the FO in 12 deceased human infants of whom 10 were live-born, Naeye *et al*. demonstrated that FO closure occurring late in pregnancy did not affect development of the left heart structures but rather resulted in changes of the right heart and pulmonary vasculature^23^. A case report of an infant with late FO closure due to a aneurysmatic changes of the intra-atrial septum and mild LV hypoplasia (despite a small VSD) seems to further support this notion^24^. Schall *et al*. speculated that his observed phenotype^8^, which is mimicked by our experiments here, represents the “missing link” between the majority of reported cases with early FO closure and significant deformities of the left heart, and the few cases with late antenatal FO closure as reported by Naeye *et al*.

During human fetal development, cellular proliferation (hyperplasia) is the main mechanism of cardiac growth, while the mature heart grows through hypertrophy but has very limited capacity for hyperplasia. Accordingly, there is a replacement of preferential hyperplasia by preferential hypertrophy at some point during development^25,26^. In our study, there is supportive evidence for RV hypertrophy rather than hyperplasia, suggesting that the switch from the hyperplastic potential already occurred at 0.7 gestation. This is supported by the observation that second trimester human fetal intervention for aortic stenosis has not been shown to fully prevent LV hypoplasia^27^. Conceivably, the time-window for preferential hyperplastic remodeling in the human may be prior to the earliest currently feasible fetal intervention.

The group of fetuses with LV hypoplasia who are most likely to benefit from fetal interventions and undergo hyperplastic remodeling are those with a “borderline” (i.e. moderately hypoplastic) LV. Our study in fetal sheep has produced the phenotype of LV hypoplasia which matches the findings in the moderate clinical spectrum of human HLHC, and represents a significant proportion of patients. This large mammalian model of LV hypoplasia induced without prior direct surgery or scarring on the fetal heart, comparable to human in size and circulatory pattern, has the potential to be used in investigation of disease pathophysiology and devising treatment strategies, eg. maternal hyperoxygenation^28^.

Our study has limitations such as the small number of animals as well as a relatively large range of gestational age at the time of the intervention. Anchoring the occluder within the stent and positioning the occluder discs to obliterate FO blood flow was difficult. However, animal models to study the effect of FO occlusion or obstruction in the fetus are rare^27^, and our technique of in-utero cardiac catheterisation allows us to manipulate the FO and study the effects of such an intervention during fetal development. In this study, fetuses with occluded FO were compared to aged-matched control fetuses which did not undergo the experimentation. In our published experimental data in the fetal sheep on the procedures of percutaneous transhepatic access both with and without positioning of a stent at the FO^16–18^, we did not observe any compromised haemodynamics on the post-procedural echocardiography, or any altered LV development on examination of the hearts at term birth. An additional sham group of fetuses was therefore not performed in the interest of limiting animal sacrifice.

## Conclusion

We have established a large mammalian model of LV hypoplasia mimicking moderate HLHC, using percutaneous fetal cardiac catheterisation and novel interventional techniques to occlude the fetal foramen ovale, without any direct surgery or scarring on the fetal heart. The model can potentially be applied in future studies to understand the pathophysiology of and develop new therapies for LV hypoplasia.

## Methods

All procedures in this study were performed using sterile surgical techniques and in accordance with the guidelines of the Australian Code of Practice for the Care and Use of Animals for Scientific Purposes, established by the National Health and Medical Research Council of Australia. The Monash University Animal Ethics Committee approved this study.

### Animals and preparation

Fetal lambs at 104–117 days gestation (Merino/Border-Leicester cross, term 147 days) were used. All ewes were brought into the animal house 1 week before the procedure for acclimatisation. The ewes were initially anaesthetised with an intravenous injection of sodium thiopentone (20 mg/kg; Pentothal), and then placed supine, intubated and ventilated. Anaesthesia was subsequently maintained using 2% Isoflurane in O_2_. The ewe received maintenance intravenous fluid (130 ml/h) of 0.18% saline + 4% glucose for the duration of anesthesia. Antibiotics (ampicillin 500 mg and gentamicin 80 mg) were administered during the procedure and repeated daily for 3 days.

Ultrasound (Voluson 730 Expert, GE Healthcare, Chicago, IL, USA) was used to determine the fetal number and position, and optimal access point. When required, suboptimal fetal position was managed by external manipulation of the fetus to improve access. No fetus was excluded or suspended from the procedure due to a suboptimal position. Ultrasound was used to measure the distance from the skin to the right hepatic vein (RHV) or left hepatic vein (LHV), the diameters of the RHV, LHV, inferior vena cava (IVC), FO, tricuspid valve, mitral valve, pulmonary valve and aortic valve, width of the RV and LV in end-systole, and the length of IVC between the insertion of the RHV or LHV and the right atrium (RA).

### Foramen ovale occlusion

Access to the fetal venous system was obtained using a percutaneous puncture through the maternal abdominal and uterine wall and into the fetal abdomen, under continuous ultrasound guidance as we described previously^16^. Depending on the position of the fetus and the ease of access, the RHV or LHV was selected for puncture and sheath insertion. Hepatic access and sheath insertion was achieved using either a 13.3-cm, 14 G intravenous catheter (BD Angiocath, Becton Dickinson, North Ryde, Australia) or a 4.5 F (ID) sheath (M001207020, ACCUSTICK™ II Introducer Systems, Boston-scientific, MA, USA) inserted into the fetal RHV or LHV. The catheter sheath was positioned close to the junction of the hepatic vein and IVC (Fig. 1a). Under ultrasound guidance, a 0.014-inch soft or stiff coronary guide wire (Hi Torque Balance Middleweight Universal, or Hi Torque Extra S’port, Abbott Vascular, Santa Clara, CA, USA), was inserted into the sheath with or without utilising a 1.8–2.6 F tapered catheter (FineCross MG, Terumo, Macquarie Park, Australia), and placed through the IVC, RA and FO, into the LA. After stable positioning of the coronary guidewire, the ensemble of a 4 F delivery catheter system loaded with a self-expandable, open-cell-design, flexible stent (8 × 12 mm Superflex DS, OptiMed, Ettlingen, Germany) was advanced over the guidewire, to position and expand the stent across the FO (Fig. 1b) as previously described^17^. The delivery catheter was then carefully withdrawn, leaving the guidewire within the LA. The position of and flow through the expanded stent were documented by 2D fetal echocardiography and colour Doppler. A 4 F Amplatzer delivery sheath was advanced over the guidewire and positioned inside the stent for anchoring an Amplatzer Duct Occluder (ADO II, 9-PDA2-04-04, 4 × 4 mm, St Jude Medical, Minnesota, USA), whereby both discs of the occluder with an expanded width of almost 10 mm occluded the stent within the atrial septum (Fig. 1c). The Amplatzer delivery system was then withdrawn. Occlusion of the stent and FO was also documented by 2D echocardiography and color Doppler. Upon removal of the transhepatic sheath, the intrahepatic portion of the 14 G intravenous catheter entry canal was embolised to reduce the risk of intra-abdominal haemorrhage in the fetus (Gelfoam^®^; Pharmacia & Upjohn Company, Michigan, USA). A video of the complete procedure is available as Supplementary Material.

In one fetus, data on myocardial tissue deformation (strain) were collected immediately before and after FO closure using 2D speckle tracking for both ventricles (Aplio 500 system, Toshiba Medical Systems Corporation, Tochigi, Japan), equipped with a 1–5 MHz curved array probe (PVT 375 BT) as we previously described^29–31^. We assessed global longitudinal peak systolic strain (LPSS) for both ventricles in the fetal lamb as previously described^29–31^. The frame rate for analysis was 60 fps. According to the consensus paper for the assessment of LPSS, endocardial strain was used to calibrate the LPSS^32^. In the offline analysis, one fetal heart cycle was selected by anatomical M-Mode. In a 3-point-analysis, an experienced operator set the endocardial borders of every cardiac cavity (endocardial tracing). This technique of speckle-tracking echocardiography offline analysis was recently described for the assessment of the atrial and biventricular strain of human fetal hearts^33^. Due to limitations of the echocardiographic console used, systolic strain rate and diastolic parameters were not analysed, as what were performed in human assessments^34^.

After recovery from the general anaesthesia, the fetuses were monitored with daily ultrasound for the first 3 days and then weekly ultrasound to check fetal well-being.

The ewes and fetuses were euthanised at 3–5 weeks after the FO occlusion, by pentobarbital sodium overdose. For twin pregnancies, only 1 fetus had the FO occlusion performed, and the twin was used as control for comparison. For singleton pregnancies, gestational age-matched fetuses were euthanised as controls for comparisons in cardiac morphology and histopathology.

At post-mortem, the fetuses were weighed. The hearts were excised with the ascending aorta and weighed. Freshly excised hearts were retrogradely perfusion-fixed via the aorta. Prior to flushing the hearts with saline, we infused heparin to prevent clotting, papaverine to dilate the cardiac vasculature, and KCl to arrest the hearts in diastole^35^. The saline infusion was followed by freshly prepared 4% formaldehyde. The fixed hearts were stored in 10% buffered formalin prior to tissue sampling.

### Cardiac morphology and histopathology

The fixed hearts were weighed after removal of connective tissue and fat. The atria were carefully dissected and the FO was examined for positions of the stent and occluder. The atria were then removed.

Each heart was assigned an arbitrary number to permit blinding to the experimental group in subsequent analyses. The volume of each ventricular chamber was measured by amount of fluid required to fill it up to the level of the atrioventricular valves. The fixed hearts were then transversely sliced at 5 mm thickness, with the first slide at the level of the aortic valves and the second slide at the level of the atrioventricular valves.

#### Measurement of ventricular wall thickness and lumen area

The annuli of the aortic, mitral and tricuspid valves were measured in the heart slices. Morphometric measurements were made on digital images of transverse sections of the heart (as described above) using image analyser (Image-Pro Plus Version 6.0, Media Cybernetics, USA). Images were used to determine septal and ventricle wall thickness and area, and atrioventricular valvular areas^35^. The wall volumes of the ventricles and septum were determined using the Cavalieri principle^36^.

#### Heart tissue sampling

Due to the haemodynamic differences between the ventricles before and after birth, the LV and RV were analysed separately. The LV was sampled together with the adjacent septum (S) as the septum is structurally similar to the left ventricular wall.

Sampling of heart tissue for stereologic analyses was performed using a smooth fractionator approach^37^. The selected samples of RV and LV + S were embedded in either glycolmethacrylate or paraffin.

#### Cardiomyocyte number

Glycolmethacrylate blocks were sectioned at 20 µm and every 10^th^ section stained with hematoxylin in a 1000 W microwave oven at 50% power for 2–4 min. This ensured adequate nuclear staining throughout the sections. Cardiomyocyte nuclei number was estimated using an optical disector–fractionator approach; we used a light microscope (Olympus BX4, Japan) coupled with a motorised stage and a z-axis sensor. Every second section was systematically sampled and projected onto a computer screen. An unbiased counting frame (329.6 mm^2^) was superimposed on the image using C.A.S.T (Computer Aided Stereological Toolbox) software (Olympus, Denmark). Nuclei were counted when they came into clear focus in the disector area (so long as no part intersected the forbidden lines) within a 10 mm depth in the middle of the section. The total numbers of cardiomyocyte nuclei in the RV and LV + S were estimated by multiplying the number of nuclei counted stereologically by the inverse of all sampling fractions^35^.

#### Measurement of cardiomyocyte size

Paraffin-embedded blocks of the RV and LV + S were cut at 40 µm. A 5 µm section from each block was cut initially for haematoxyline stain and examination to ensure longitudinal orientation of the cardiomyocytes in the paraffin block, and re-embedding was performed if required. Three paraffin sections from each of RV and LV + S of each animal were stained with 10 mg/L of wheat germ agglutinin-Alexa Fluor 488 conjugate (Molecular Probes Invitrogen, USA) and 1:5000 YOYO-3 (Molecular Probes Invitrogen). Wheat germ agglutinin-Alexa Fluor 488 conjugate stains the cell membranes and YOYO-3 stains the nucleus. We used a Leica SP5 broadband multi-photon confocal microscope (Leica, Germany) with a Spectra-Physics MaiTai Ti:Sapphire multi-photon source (Spectra-Physics, Newport Corporation, USA) to examine cardiomyocyte nuclearity. Volocity Version 5 software (Perkin Elmer, UK) was used to visualise and measure the cardiomyocyte longitudinal cross-sectional area, by tracing the boundaries of cardiomyocytes in which the nuclei could be seen in the center. One hundred mononucleated and 100 binucleated cardiomyocytes were measured in each RV and LV + S for each animal^35,36^.

#### Quantification of interstitial collagen

Paraffin-embedded blocks of RV and LV + S were sectioned at 5 µm and stained with picrosirius red after pre-treatment with phosphomolybdic acid. Three sections from each of RV and LV + S were used and the percentage of collagen within the interstitium was quantified using image analysis (Image-Pro Plus Version 6.0, Media Cybernetics)^35^.

### Statistical analysis

Data is expressed as mean ± SD. All data were compared between the fetuses with FO occlusion and the controls using an independent sample two-tailed t-test for parametric data, or Mann-Whitney U test for non-parametric data. Ultrasonographic measurements in the fetal hearts before the FO occlusion were compared with those taken 30 mins after the FO occlusion using the paired t-test. Statistical significance was defined as a p-value of <0.05.

The datasets generated during and/or analysed during the current study are available from the corresponding author on reasonable request.

## Supplementary information

Supplementary Video.

Supplementary Table and legend for the Supplementary video.
