# Supplementary material for: Induction of left ventricular hypoplasia by occluding the foramen ovale in the fetal lamb
Source: Sci Rep. 2020 Jan 21;10:880. doi: 10.1038/s41598-020-57694-4 (PMC6972793; doi:10.1038/s41598-020-57694-4)
Supplement: Supplementary file 2 — Supplementary Table and legend for the Supplementary video. [file 41598_2020_57694_MOESM2_ESM.docx]

**Induction of left ventricular hypoplasia by occluding the foramen ovale in the fetal lamb**

*Flora Y. Wong PhD, Alex Veldman MD, Arun Sasi MD, Mark Teoh MBBS, Andrew Edwards MD, Yuen Chan MBBS, Oliver Graupner, Christian Enzensberger, Roland Axt-Fliedner, Mary Jane Black PhD, and Dietmar Schranz MD

SUPPLEMENTARY INFORMATION

**Legend for the supplementary video file:** The complete fetal catheterization procedure including the percutaneous transhepatic venous puncture, advancement of guidewire to the foramen ovale and left atrium, insertion of the stent across the foramen ovale with Doppler to show flow through the stent, and anchorage of the occluder within the stent. With occlusion of the foramen ovale, the left ventricle collapsed. On withdrawal of the sheath from the hepatic vein, gelform was injected to embolize the sheath entry canal in the fetal liver.

**Supplementary Table S1**

| **Values are mean (SD)** | **Control (n=9)** | **Partially occluded foramen ovale (n=5)^$^** | **Occluded foramen ovale (n=6)** | **P** |
| --- | --- | --- | --- | --- |
| Body weight, g | 5233 (65) | 4828 (70) | 4853 (48) | 0.39 |
| Weight of both ventricles, g | 23.61 (5.7) | 23.5 (2.9) | 21.13 (3.2) | 0.55 |
| Ratio of ventricle to body weight | 4.5 (0.7) x10^-4^ | 4.9 (0.4) x10^-4^ | 4.3 (0.4) x10^-4^ | 0.29 |
| Aortic valve cross-diameter, mm | 8.9 (1.6) | 6.8 (1.5)*# | 4.8 (1.0)** | **<0.001** |
| **Atrio-ventricular valve area, cm^2^** |  |  |  |  |
| Mitral valve (MV) | 1.17 (0.45) | 1.23 (0.40) | 0.72 (0.31) | 0.08 |
| Tricuspid valve (TV) | 0.85 (0.37) | 1.39 (0.50) | 0.83 (0.40) | 0.06 |
| MV/TV ratio | 1.44 (0.28) | 0.94 (0.27)** | 0.88 (0.09)** | **<0.001** |
| **Ventricular chamber volume, ml** |  |  |  |  |
| LV | 2.28 (1.53) | 1.88 (1.17) | 0.92 (0.72) | 0.15 |
| RV | 2.27 (1.45) | 3.1 (1.41) | 2.33 (1.57) | 0.58 |
| LV/RV ratio | 1.00 (0.14) | 0.75 (0.52) | 0.39 (0.14)** | **0.007** |
| **Ventricle weight, g** |  |  |  |  |
| LV+S | 12.97 (3.34) | 12.72 (0.78) | 11.08 (2.25) | 0.39 |
| RV | 6.61 (1.77) | 7.77 (1.47) | 8.08 (1.43) | 0.20 |
| LV+S/RV ratio | 1.98 (0.18) | 1.68 (0.31) | 1.37 (0.12)** | **0.002** |
| **Ventricle wall volume, cm^3^** |  |  |  |  |
| LV+S | 13.98 (3.77) | 13.72 (1.11) | 11.00 (0.85) | 0.07 |
| RV | 6.44 (1.33) | 7.41 (1.15) | 7.57 (0.91) | 0.16 |
| LV+S/RV ratio | 2.16 (0.24) | 1.88 (0.29)# | 1.47 (0.16)** | **<0.001** |

^$^Of the 7 fetuses with partial FO occlusion due to suboptimal position of the occluder, 2 fetuses also had a mitral valve leaflet attached to the distal occluder disc resulting in very dilated left hearts, and were excluded from the morphology measurements.

P: p-value using one way ANOVA for parametric data, or ANOVA on Ranks for non-parametric data.

*P<0.05, **P<0.01: Post-hoc comparison with Control.

#P<0.05: Post-hoc comparison with Occluded foramen ovale.
